# Surface Defect-Extended BIM Generation Leveraging UAV Images and Deep Learning

**DOI:** 10.3390/s24134151

**Published:** 2024-06-26

**Authors:** Lei Yang, Keju Liu, Ruisi Ou, Peng Qian, Yunjie Wu, Zhuang Tian, Changping Zhu, Sining Feng, Fan Yang

**Affiliations:** 1 Key Laboratory of Urban Land Resources Monitoring and Simulation, Ministry of Natural Resources, Shenzhen 518034, China; 2118310009@stmail.ntu.edu.cn; 2 School of Transportation and Civil Engineering, Nantong University, Nantong 226019, China; 2233310004@stmail.ntu.edu.cn (Y.W.); 2333310011@stmail.ntu.edu.cn (Z.T.); 3 Nantong Key Laboratory of Spatial Information Technology R&D and Application, Nantong University, Nantong 226019, China; 2221110020@stmail.ntu.edu.cn (K.L.); 2321110065@stmail.ntu.edu.cn (R.O.); 4 College of Geographic Science, Nantong University, Nantong 226019, China; 2108110166@stmail.ntu.edu.cn (C.Z.); 2221110182@stmail.ntu.edu.cn (S.F.)

**Keywords:** BIM, surface defect detection, UAV, deep learning, texture mapping

## Abstract

Defect inspection of existing buildings is receiving increasing attention for digitalization transfer in the construction industry. The development of drone technology and artificial intelligence has provided powerful tools for defect inspection of buildings. However, integrating defect inspection information detected from UAV images into semantically rich building information modeling (BIM) is still challenging work due to the low defect detection accuracy and the coordinate difference between UAV images and BIM models. In this paper, a deep learning-based method coupled with transfer learning is used to detect defects accurately; and a texture mapping-based defect parameter extraction method is proposed to achieve the mapping from the image U-V coordinate system to the BIM project coordinate system. The defects are projected onto the surface of the BIM model to enrich a surface defect-extended BIM (SDE-BIM). The proposed method was validated in a defect information modeling experiment involving the No. 36 teaching building of Nantong University. The results demonstrate that the methods are widely applicable to various building inspection tasks.

## 1. Introduction

With the continuous development and expansion of cities, it has become increasingly difficult for older towns to accommodate the infrastructure needs associated with social and economic development. Urban renewal and renovation are gradually being regarded as social projects and have received widespread attention [1,2,3,4]. In urban renewal and renovation projects, building surface defects directly reflect the reduction in structural durability. Therefore, surface defect inspection is an indispensable aspect of building safety appraisal tasks [5,6,7]. Modern building architecture typically uses multi-floor designs, and due to the high elevation, it becomes difficult to manually obtain defect information from upper floors.

The development of unmanned aerial vehicle (UAV) technology provides more possibilities for building information acquisition in the AEC/FM field. UAVs are low cost, highly efficient, and are widely used in building surveying and 3D reconstruction. With the powerful structure-from-motion (SfM) algorithm [8,9,10], one or more sets of drone image sequences can be employed to generate a three-dimensional model of a scene. Presently, several commercial 3D reconstruction applications are available, including Context Capture, PIX4Dmapper, and Reality Capture, offering high-quality real-3D-model production services. Some studies have started to use UAVs for defect inspection. However, the accuracy of traditional defect detection methods is low, which makes it difficult to meet building inspection requirements. With the development of artificial intelligence technology, some scholars have used deep learning methods for defect detection from images. However, these studies simply analyzed the defect information in images. None of them integrated the defect inspection information into BIM models, which is highly convenient for digitalizing archives and transfers. The key problem for mapping the results of building surface defect inspection results onto the surface of BIM models is the coordinate difference between UAV images and BIM models. Therefore, improving defect detection accuracy based on deep learning methods and studying mapping methods from image coordinates to the BIM project coordinate system are highly important.

In this study, a framework for creating an SDE-BIM model that leverages UAV images and deep learning is proposed. The contributions of this study are twofold: (1) a defect inspection dataset is created using UAV images, and a deep learning-based method coupled with transfer learning is used to detect defects accurately; (2) a texture mapping-based defect parameter extraction method is proposed to implement mapping from the image U-V coordinate system to the BIM project coordinate system.

The remainder of this paper is organized as follows. Relevant research on architectural modeling and defect modeling is reviewed in Section 2. The proposed framework for producing building surface defect information is introduced in Section 3. The experiment on the No. 36 teaching building of Nantong University is described in Section 4. The discussion is presented in Section 5, and finally, the conclusions are presented in Section 6.

## 2. Related Works

In the realm of AEC/FM, 3D reconstruction technology is widely used in building model production and BIM object reconstruction [11,12]. Currently, image processing and deep learning methods [13,14,15] are extensively used in object detection and semantic segmentation. An endless stream of new methods makes defect inspection based on deep learning image processing possible [16,17]. In urban renewal projects, automatically and efficiently monitoring and assessing building health conditions is indispensable. Integrating BIM models with building surface defect inspection is gaining increasing attention. Related research endeavors are reviewed in this section.

The traditional nondestructive inspection methods [18] for buildings use modern technology and manual operation of equipment, and study the various properties of building defects by detecting changes in internal structural abnormalities or reactions caused by various defects such as sound, light, magnetism, heat, and electricity. Traditional methods have many limitations, such as high-precision instruments being easily affected by environmental factors, dangerous inspection locations, and radiation damage to the human body caused by radiographic inspection.

In recent years, the rapid development of drone technology [5,6,19] and wall-climbing robots [20] has provided powerful building inspection tools. Engineers can easily control drones by installing high-definition cameras on the gimbal to obtain information about the development of defects in high buildings and hazardous areas. Through UAV oblique photogrammetry, real data of existing buildings can be obtained, resulting in the generation of 3D point clouds and parameterized BIM models [21,22,23]. However, the large quantity of defect data acquired through drones requires manual processing and lacks automated detection methods.

With the development of artificial intelligence, deep learning has promoted further improvement of the automated detection technology. Unlike traditional manual nondestructive inspection models, semantic segmentation models that learn many defect features can replace humans in performing defect detection tasks. Defects can be distinguished at the pixel level [24] through these types of computer-executed defect inspection tasks, which are more accurate than manual detection. Moreover, real-time inspection can be achieved by training lightweight models [25]. At present, many excellent image segmentation architectures are available, such as classic U-Net [26], FCN [27], GoogLeNet [28], cutting-edge K-Net [29], PIDNet [30], and mask2former [31]. However, a significant drawback of the semantic segmentation models is the inability to obtain the geometric feature parameters of the defects. Typically, after predicting the defect area through the model, traditional image processing methods are used to calculate these feature parameters.

After automating defect inspection, a new problem arises because the obtained defect information is stored in a large quantity of images and is difficult to manage. Some scholars have considered expanding the information exchange function of BIM models and integrating defect information with BIM models [32]. In this way, integrating BIM and deep learning, and generating defect-extended BIM have become new development trends. Scholars have performed research on this topic. Pantoja et al. [33] proposed an end-to-end framework for generating level-of-detail 3 (LOD3) damage-augmented digital twins. The LOD3 model is generated based on SfM, and deep learning methods are used to segment cracks on the building surface. The defect information is mapped onto the surface of the LOD3 model using the simulated light method, thereby generating a 3D building model containing defect information. However, the LOD3 model obtained through this method exhibits limited semantic information and defect types, leading to inadequate building information sharing. Kopsida et al. [34] employed KinectFusion for the 3D reconstruction of buildings, achieving registration between as-built models and as-planned BIM models by estimating camera poses and utilizing the iterative closest point (ICP) algorithm. Liu et al. [35] aligned the real camera pose coordinates with the virtual camera coordinates in the BIM model and achieved registration between the 3D reconstruction model and the BIM model. In Chen’s study [36], a registration method based on real images and BIM-projected images was proposed. Buildings and irrelevant backgrounds are differentiated using masks, allowing the extraction of the structure of interest (SOI) for defect inspection. In another of Chen’s studies [37], the method described in reference [36] was utilized for coarse registration. After defect inspection using U-Net, the camera pose information of the images was utilized to project onto the surface of the BIM model, integrating defect information with BIM.

Analyzing existing research shows that the defect inspection quality and the alignment accuracy of different coordinate systems [38,39] are vital to generating defect-extended BIMs. We have also researched these two key areas in Section 3.

## 3. Proposed Methods

**Figure 1:**
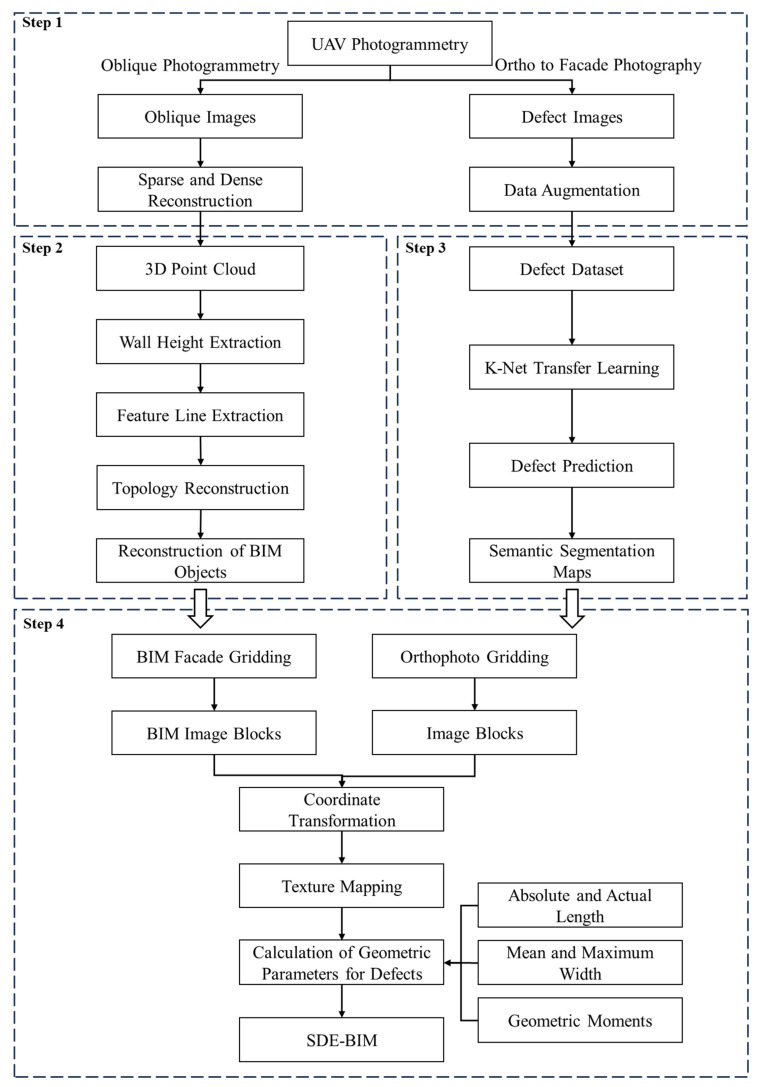
Framework for creating the surface defect-extended BIM.

The framework process for constructing a surface defect-extended BIM model, as depicted in Figure 1, comprises four major tasks. (1) UAV image acquisition and 3D reconstruction: The UAV images are captured through oblique photography and orthophotography. The oblique images are used for 3D reconstruction and the orthophotos are used for defect dataset production after data augmentation. (2) Generation of BIM model from dense point cloud: In this stage, building point clouds are generated from UAV oblique images captured during field work, and the BIM objects are subsequently produced from the point clouds. (3) Defect inspection using deep learning: The K-Net neural network is selected for defect inspection. The pretrained K-Net model is used for transfer learning, the defect dataset is augmented to enhance the generalization ability. (4) Texture mapping-based SDE-BIM creation: The texture of the building surface defects is mapped onto the surface of the reconstructed BIM model using WebGL technology. The geometric parameters of the surface defects are calculated from the orthophotos.

### 3.1. UAV Image Acquisition and 3D Reconstruction

**Figure 2:**
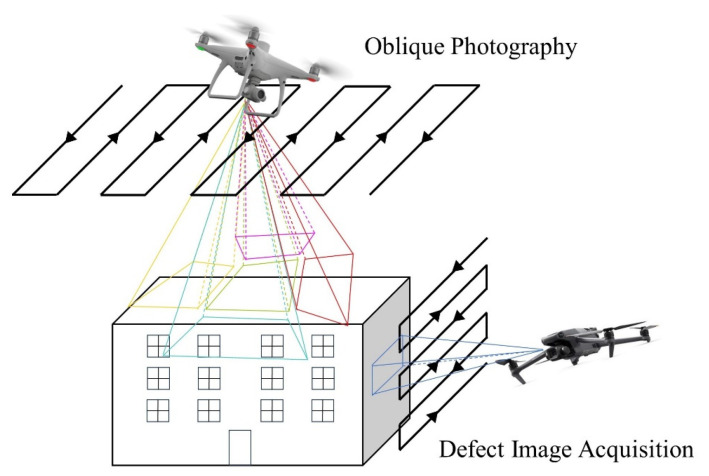
Five-directional flight for oblique photography and orthophotography for defect image acquisition.

In this research, drone images are used for generating dense point clouds and conducting defect inspection. The task of capturing drone images is divided into two subtasks for different purposes: oblique photogrammetry for 3D reconstruction to generate point clouds and ortho-to-facade orthophotography for defect detection. The UAV oblique photogrammetry adopts a five-directional flight mode, which is automatically executed after planning the mission. The orthophotography task uses drones with higher photography accuracy to manually fly close to the facade (within 5 m) and obtain clear images. As Figure 2 shows, the five-directional flight mode can capture rich architectural images at higher altitudes, making the 3D reconstruction results more precise; however, obtaining defect information about the facade is difficult. In contrast, it is difficult to capture image groups containing many corresponding points with facade orthophotography due to its small image distance. However, it can capture fine defect images, which compensates for the disadvantage of five-directional flight.

After the field work, unqualified image data are deleted by checking the clarity, reflectivity, and presence of obstacles. Qualified drone oblique images are used for 3D reconstruction based on SfM to generate original point clouds, and facade orthophoto images are used to produce defect datasets.

### 3.2. Generation of BIM Models from Dense Point Clouds

Aerial triangulation is conducted after rejecting images with errors, blurriness, and fewer homologous points from the original set of UAV oblique images. The point cloud of the target building is cropped out of the reconstructed 3D scene and subjected to filtering and denoising to ensure the accuracy and efficiency of architectural structural parameter extraction. In this paper, the height of the building and wall line features are indispensable structural parameters that must be extracted from the point cloud.

#### 3.2.1. Wall Height Extraction

**Figure 3:**
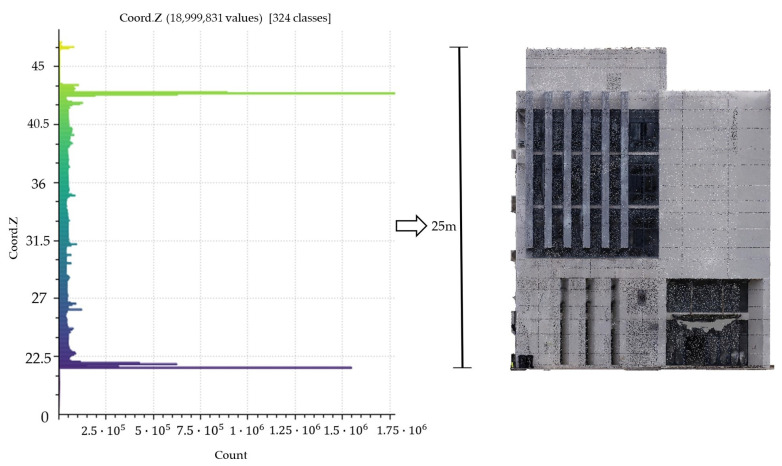
Building height estimation based on Gaussian clustering. The left part is a point height histogram of a building. The y-axis references the heights of the building dense point clouds, and the x-axis refers to the number of points. The points distributed at the top (in green) and bottom (in purple) elevations is the highest due to the hollow structure of the point cloud.

The height of the building is estimated by the Gaussian clustering method [40]. As shown in Figure 3, the principle of height estimation involves clustering the building point cloud of the building according to elevation (along the Z-axis), where each elevation corresponds to a certain quantity of sample points. The quantity of sample points distributed at the top (in green) and bottom (in purple) elevations is the highest due to the hollow structure of the point cloud. This results in a bimodal distribution in the elevation histogram, where the absolute distance between the two peaks corresponds to the actual building height.

#### 3.2.2. Feature Line Extraction

**Figure 4:**
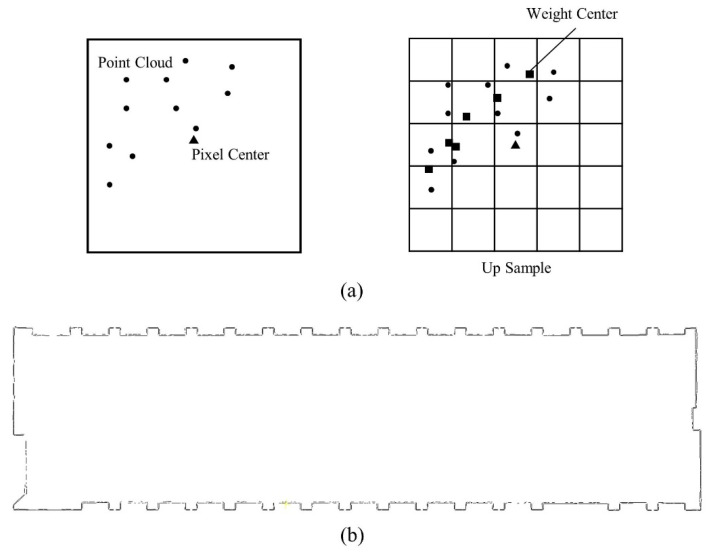
E-LSD algorithm for feature line extraction. (**a**) The center calculated by traditional rasterization methods and the weight center calculated in this study; (**b**) example of feature line extraction.

Using the entire dense point cloud when reconstructing BIM objects is time consuming due to the massive and complex building cloud characteristics. Considering that buildings are composed of walls with distinct line features, point cloud slice generation can be employed to simplify the wall modeling problem into a line feature extraction problem. However, existing methods for feature line extraction are still not sufficiently mature in terms of extraction accuracy and topological consistency. The efficiency is relatively low if the features are calculated directly based on points. Rasterization can improve computational efficiency when the pixel size is small enough and ensure sufficient accuracy. Common image edge detection algorithms, such as the Canny operator and the LSD algorithm, utilize gradient information to calculate edges. However, for rasterized point cloud slices, using edge gradient information alone cannot accurately extract the centerline of the slice, leading to a decrease in the precision of the extracted line features. Therefore, a feature line detection algorithm based on eigenvector calculation (E-LSD) is proposed in this study (as shown in Figure 4), which replaces the gradient direction with the eigenvector direction, enabling accurate slice segment detection. This method ensures geometric precision in extracting line segments by utilizing smaller pixel sizes. The specific process is detailed in Appendix A.

#### 3.2.3. Topology Reconstruction

The feature lines extracted in the last step are incomplete (including broken lines and incorrectly intersected lines) [41]. The feature line segments in set L exhibit three types of positional relationships: parallel, collinear, and intersecting (perpendicular). Therefore, repair and fusion are indispensable for structural topology reconstruction. First, the midpoint of each line segment $L_{i}$ in the set L is calculated to construct a Delaunay triangulation net. The neighbors M of the current line segment $L_{i}$ are located by examining each edge in the Delaunay triangulation net based on the positional relationship between the current segment and its neighboring segments; the repair and fusion methods are executed as follows:

(1) Traverse each line segment $L_{i}$ and consider its neighbors $L_{j}$ and $L_{k}$. When $L_{i}$ is perpendicular to $L_{j}$ and parallel to $L_{k}$, the direction of $L_{i}$ is adjusted to align with the direction of $L_{k}$.
(2) Traverse each line segment $L_{i}$ and obtain the neighboring line segment $L_{j}$ within its neighborhood $M_{i}$. If the line segment $L_{i}$ is collinear with $L_{j}$ and there are no closer neighbors between $L_{i}$ and $L_{j}$, the line segment is added to the repair set $L_{repair}$.
(3) Traverse each line segment $L_{i}$ and obtain the neighboring line segments $L_{j}$ within its neighborhood $M_{i}$. If $L_{i}$ is perpendicular to $L_{j}$, calculate the intersection point between $L_{i}$ and $L_{j}$, and add the line segment to the repair set $L_{repair}$.

**Figure 5:**
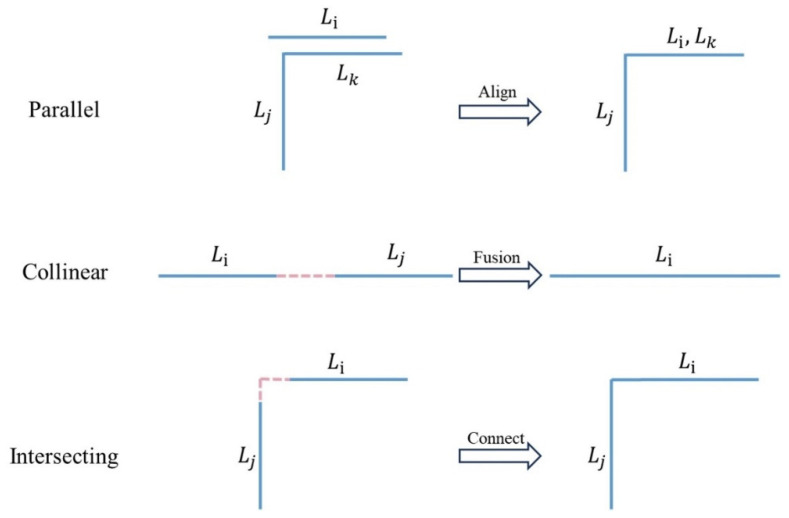
Visualization of the topology reconstruction process.

After repairing all line segments, traverse each line segment $L_{i}$ and obtain the neighboring line segments $L_{j}$ within its neighborhood $M_{i}$. When the line segment $L_{i}$ is connected to its neighbor $L_{j}$, the neighboring and current line segments are merged into a polyline. Finally, the polyline collection PL is obtained. The process of topology reconstruction is visualized in Figure 5.

#### 3.2.4. Reconstruction of BIM Objects

**Figure 6:**
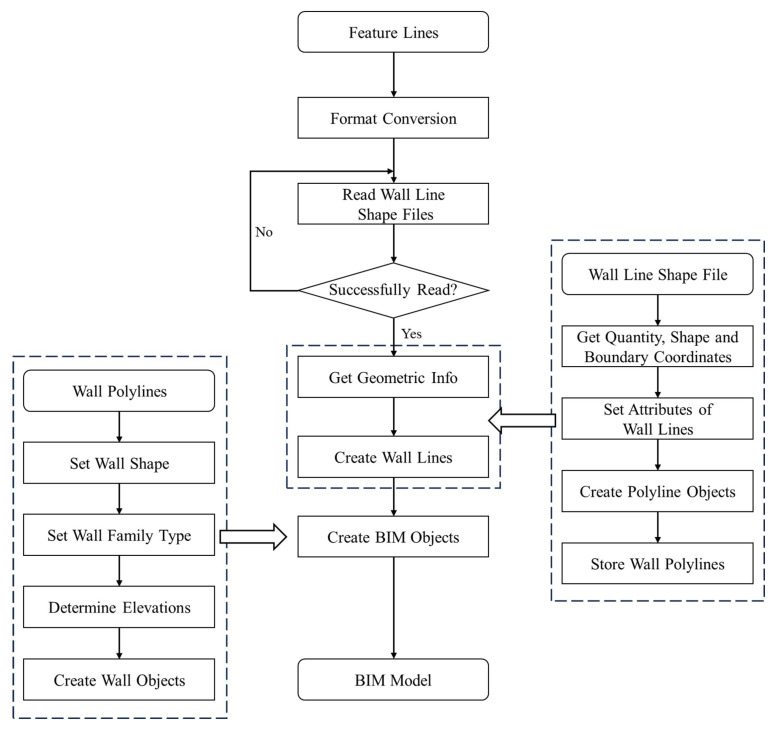
Workflow for automated reconstruction of BIM wall objects.

After obtaining the structural parameters, the BIM objects are automatically reconstructed using the developed Revit plugins proposed in our previous work [42], thus obtaining the BIM model of the target building. The workflow for automatically generating BIM wall objects based on the extracted structural parameters is shown in Figure 6. Floors, ceilings, and other structures are similarly added to the BIM model.

### 3.3. Defect Detection Using Deep Learning

#### 3.3.1. Selection of Deep Neural Network

**Figure 7:**
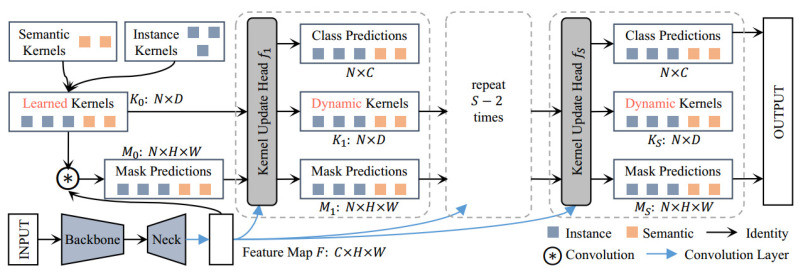
Framework for the K-Net segmentation algorithm [29].

In semantic segmentation tasks, convolutional kernels identify and group pixels with similar characteristics. Based on semantic segmentation, instance segmentation and panoramic segmentation usually require more intricate frameworks to distinguish different instance objects, resulting in fragmentation in different image segmentation tasks. To unify semantic segmentation, instance segmentation, and panoramic segmentation, the K-Net framework [29] deviates from the detection paradigm before segmentation in Mask-RCNN [43], providing a unified, simple, and effective framework. During the training process, the images are divided into groups using static kernels that have already been learned, and then, iteratively improved. The image is divided based on the features in the divided groups, as shown in Figure 7. First, a set of static kernels $K_{0}$ is convolved with feature map *F* to obtain the mask prediction $M_{0}$. Then, the feature map *F*, the learned static kernel $K_{0}$, and the mask prediction $M_{0}$ are taken as inputs, the classification prediction, dynamic kernel $K_{1}$, and updated mask prediction $M_{1}$ are obtained through $f_{1}$. Finally, the above steps are repeated to continuously obtain updated classification predictions, dynamic kernels, mask predictions, and the final image segmentation results. The K-Net framework uses a set of convolutional kernels to generate a mask. Different convolutional kernels generate masks for different categories, and segmentation tasks can be performed without any additional components. End-to-end training optimization is realized while improving inference efficiency.

In this study, K-Net and UPerNet were combined for defect inspection. A pretrained model with excellent training performance on the ADE20K dataset [44] is selected for transfer learning. Its backbone network is Swin-L. K-Net demonstrates superior image segmentation accuracy and efficiency on the same training dataset compared to some classical deep learning models. When facing semantic segmentation tasks involving building surface defects, satisfactory defect inspection and segmentation results can be obtained. Therefore, the pretrained K-Net model is utilized in this article as the foundational model for transfer learning in the semantic segmentation of building surface defect information.

#### 3.3.2. Augmentation of the Defect Dataset

**Figure 8:**
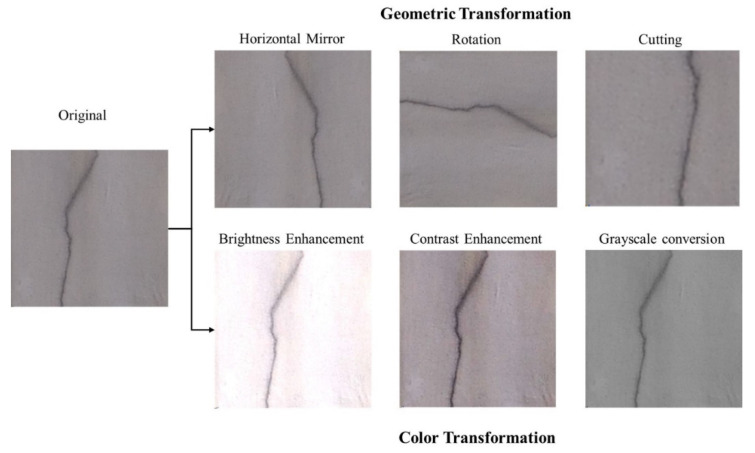
Data augmentation approaches.

Due to the limited number of original images in the dataset, direct training may result in unsatisfactory models. Dataset augmentation on the original images in the dataset is conducted to generate more images based on the existing training samples to learn as many features as possible and improve the generalization ability of the new model. In our research, geometric and color transformations are used for data augmentation (Figure 8).

#### 3.3.3. Transfer Learning and Evaluation

Transfer learning leverages pretrained models on large-scale datasets as a universal feature extractor, and then, fine-tunes them on tasks in new fields. The pretrained models share low-level visual features such as edges, gradient changes, shape, geometric changes, and brightness changes in different semantic segmentation training processes, and the improvement effect on training new models is relatively significant. Relatively few of the famous open-source datasets contain building defects. The cost of training a model from scratch is relatively high. Therefore, using transfer learning methods for defect detection is a good choice.

In supervised learning, the confusion matrix (Table 1) and its indicators are typically employed to evaluate the training process. The precision, recall, accuracy, IoU, Dice coefficient, and F score are frequently used. The calculation formulas for these single indicators are listed in Table 2. Unlike other indicators, IoU represents the ratio of the intersection area and union area between the annotated region and the validation region (as shown in Formula (1)). The closer the IoU value is to 1, the greater the similarity between the semantic segmentation region range and the annotation range of the model, which means that the model performs better.

(1) $$IoU=\frac{Area\;of\;Intersection}{Area\;of\;Union}$$

Typically, in multi-object semantic segmentation tasks, average indicators of the above-mentioned indicators, such as the mIoU, mAcc, and mRecall, are also utilized as references. These average indicators reflect the semantic segmentation quality of the trained model on the entire dataset.

**Table 1:** Confusion matrix.

Confusion Matrix		Prediction	
		Positive Samples	Negative Samples
**Ground Truth**	**Positive Samples**	True Positive (TP)	False Negative (FN)
	**Negative Samples**	False Positive (FP)	True Negative (TN)

**Table 2:** Evaluation indicators for deep learning.

Indicators	Formula	
Precision	$Precesion=\frac{TP}{TP+FP}$	(2)
Recall	$Recall=\frac{TP}{TP+FN}$	(3)
Accuracy	$Accuracy=\frac{TP+TN}{TP+TN+FP+FN}$	(4)
Dice Coefficient	$Dice=\frac{2\times TP}{TP+FP+TP+FN}$	(5)
F score	$Fscore=(1+\alpha^{2})\frac{precision\times recall}{\alpha^{2}\times precision+recall}$	(6)

### 3.4. Texture Mapping-Based SDE-BIM Creation

**Figure 9:**
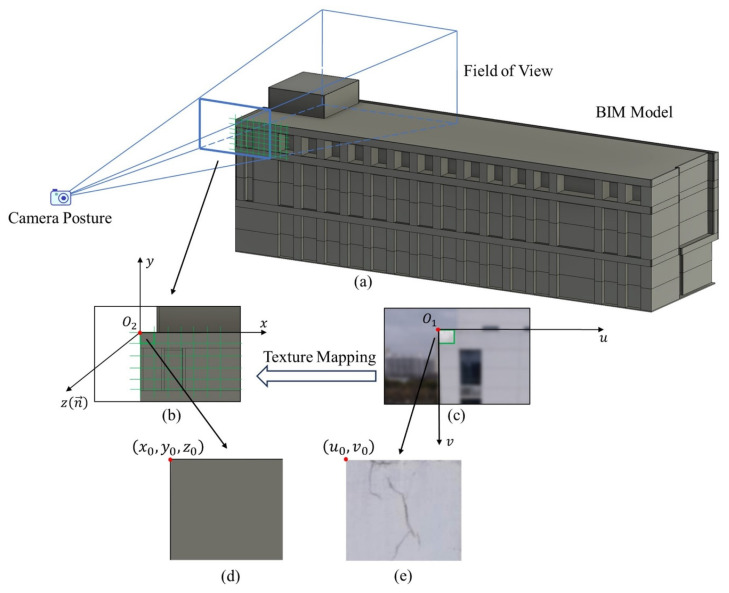
Illustration of the proposed texture mapping method. (**a**) Gridding of the BIM facade and virtual FOV; (**b**) a composite image for BIM; (**c**) a block of an image in a real-world photo which is mapped onto the square block in the BIM grid; (**d**) the square in BIM; (**e**) an image block from real-world image mapping to the square in BIM.

In this section, a texture mapping-based method is proposed to map defect information onto the surface of BIM models. In the process of using UAVs to obtain photos of building facades containing defects, drones should use the ortho-to-facade method to obtain photos. A pinhole model can be used to describe the camera’s imaging process. As is shown in Figure 9a, a virtual camera is used to simulate the camera’s imaging process. Different from taking photos from buildings in the real world, the object being photographed is a BIM model. When the camera is placed at the actual location where the drone takes photos and the same focal length is used, a composite image of the same size as the real photo will be obtained (Figure 9b). Considering the accuracy and quality of defect inspection needed to be guaranteed, a 1m×1m grid was used to crop the BIM facade into blocks. The square area in Figure 9b where the blocks are located corresponds to the pixels of area in Figure 9c in the real photo. A texture mapping method is then used to obtain a square image, as shown in Figure 9e. The point $\left(u_{0},v_{0}\right)$ in the U-V coordinate system can be transformed into the BIM project coordinate system. Assume that the original point in the project coordinate system has been transformed to $\left(x_{0},y_{0},z_{0}\right)$. The X-axis is along the direction of the BIM wall centerline. The Z-axis is along the normal vector of the BIM wall surface. The coordinates of BIM point *p* $\left(u,v\right)$ in the block can be calculated as follows:

(7) $$\left\{\begin{array}{l} x=x_{0}+u\times S_{p}\\ y=y_{0}-v\times S_{p}\\ z=z_{0} \end{array}\right.$$

where $\left(u,v\right)$ is the known pixel coordinates in the U-V system and $S_{p}$ represents the pixel size. The proposed method achieves the texture mapping of the initial grid block and the texture mapping of the entire facade can be completed through the corner coordinate transfer of the blocks. By studying the mapping relationship between the image U-V coordinate system and the BIM system, the texture mapping from real images to BIM models can be realized. The texture mapping of the remaining facades follows the same steps.

Additionally, the geometric parameters of the defects can be calculated based on the photos of the facades obtained from texture mapping. In this article, the length, width, and geometric moments are important indicators for describing the conditions of cracks.

(1) The length of the cracks

Crack length can reflect the damage condition of buildings and is one of the most direct damage evaluation indicators. As the crack length increases, the probability of structural damage to the building increases. However, it is difficult to calculate the length of cracks directly from crack images. In this article, skeletonized cracks are used to calculate the width instead of the original crack width. The crack length is divided into the absolute and actual lengths according to the different crack characteristics. The specific calculation method is as follows:

(8) $$L_{absolute}=\sqrt{{\left(x_{n}-x_{1}\right)}^{2}+{\left(y_{n}-y_{1}\right)}^{2}}$$

where $L_{absolute}$ is the Euclidean distance between the pixel coordinates of the first and last crack endpoints in the image, with the bending condition not considered. $\left(x_{1},y_{1}\right)$ and $\left(x_{n},y_{n}\right)$ (n=1, 2,⋯n) are the pixel coordinates of the starting and ending points of the crack skeleton line, respectively.

(9) $$L_{actual}=\sum_{i=1}^{n}\sqrt{{\left(x_{n+1}-x_{n}\right)}^{2}+{\left(y_{n+1}-y_{n}\right)}^{2}}$$

Typically, cracks bend. In Formula (9), $L_{actual}$ is defined as the total length of the skeleton. The total length of the skeleton is calculated by accumulating the Euclidean distances of adjacent pixels to ensure the accuracy of the calculation results.

(2) The width of the cracks

Similar to crack length, the crack width also provides important reference information for building maintenance, inspection, and repair. Crack width includes both the mean and maximum widths.

The principle of calculating the mean crack width is to treat the crack area as a rectangle; the mean width is the ratio of the rectangular area to the actual crack length. The calculation formula is

(10) $$W_{mean}=\frac{S_{pixel}}{L_{actual}}=\frac{\sum \sum I(x,y)}{\sum_{i=1}^{n}\sqrt{{\left(x_{n+1}-x_{n}\right)}^{2}+{\left(y_{n+1}-y_{n}\right)}^{2}}}$$

where $S_{pixel}$ is the pixel area of the crack region in the binarized images.

The local crack width refers to the width of the crack at any point on the crack skeleton, and the maximum width is the maximum value of the local width. Extracting the crack edge and skeleton is the first step in calculating the maximum width. Next, the pixels on the skeleton are randomly selected, and the tangent and normal lines at each point are calculated. The Euclidean distance between the pixel coordinates of the normal intersections and the edge of the crack is the local width, and the maximum width is the maximum value of the distance.

(3) The geometric moments of the cracks

Moment is an operator that describes image features and is widely used in image retrieval and recognition. The geometric moment of an image is a common geometric feature parameter used to describe the geometric shape of objects in the image and is calculated using the following formula:

(11) $$m_{ji}=\sum_{x}\sum_{y}I(x,y)\cdot x^{j}y^{i}$$

When i=j=0, $m_{00}$ is the zero-order moment that represents the pixel area of cracks, namely, $S_{pixel}$. When i+j=1, $m_{01}$ and $m_{01}$ are two components of the first-order moment. The first-order moments are used to express the centroid distribution of image objects. When i+j=2, the second-order moment has three components that express the shape and rotation conditions. In practical experiments, crack types include transverse, vertical, and oblique cracks. The second-order moment is a reliable indicator of the rotation of these three types of cracks.

## 4. Experiment

Modeling experiments were conducted on the No. 36 teaching building of Nantong University to verify the proposed method’s capability for creating the building surface defect information model.

### 4.1. Introduction for Experimental Site

**Figure 10:**
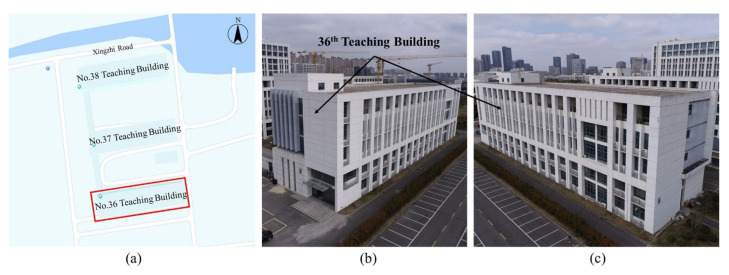
Experiment site. (**a**) The geographical location of the No. 36 building; (**b**,**c**) UAV images of the No. 36 building.

As Figure 10 shows, the No. 36 teaching building is located at the southern end of the science teaching buildings. It consists of five floors with a height of 25 m and a total floor area of 6937 square meters. It is classified as a type II multistory civil building with a level-two structural safety grade and a design service life of fifty years. The building structure comprises a reinforced concrete frame system. The exterior walls above ground level comprise 250 mm thick A5.0 autoclaved aerated concrete blocks for thermal insulation, along with M5.0 gypsum mortar for masonry.

### 4.2. Dense Point Cloud Reconstruction for the Building

During the field work stage of UAV photogrammetry, the DJI Phantom 4 RTK quad-rotor drone was equipped for capturing building images, and the DJI Mavic 3 quad-rotor drone was used to capture detailed images. Oblique photography executed the five-directional oblique photography task inherent in DJI UAVs, and its flight path was automatically adapted based on the flight area. The requirement for oblique photography of photo groups is to have an overlap of 70–80% to ensure sufficient homonymous points during 3D reconstruction. The flight altitude was approximately 70 m, the forward overlap rate was 80%, the side overlap rate was 70%, and the flight speed was 5 m/s. The ground resolution of the image was 0.02 m. Nine photo-control points (four as checkpoints) were set up and six target coordinates were captured. For the photo-control point, the CGS2000 coordinate system and Gauss three-dimensional zone projection were adopted; the central longitude was 121°, and the elevation system was the 1985 National Elevation Datum. When dealing with buildings with different sizes, considering flight safety and modeling quality, the altitude of oblique photography needs to be adjusted accordingly. The focal length of the camera is 8.5797 mm, the principal point coordinates are $\left(2722.5,1835.1\right)$, and the distortion coefficient is $D(K_{1},K_{2},K_{3},P_{1},P_{2})=(-0.2690,\;0.1116,-0.03260,\;0.0004,\;0.0004)$. A total of 178 photographs of building 36 were captured during the field work, of which 6 erroneous images were removed, leaving 172 images for 3D reconstruction.

**Figure 11:**
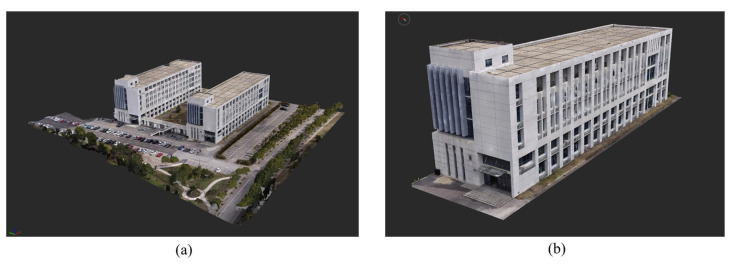
3D scene model. (**a**) Result of the 3D reconstruction; (**b**) No. 36 building, clipped form of (**a**).

During the interior work stage of the 3D reconstruction, the Context Capture 10.17 software was used to reconstruct the 3D model of the actual architectural scene, from which the dense point cloud of the No. 36 building was clipped out (as shown in Figure 11) for subsequent extraction of the building structural parameters.

### 4.3. Production of the BIM Model

**Figure 12:**
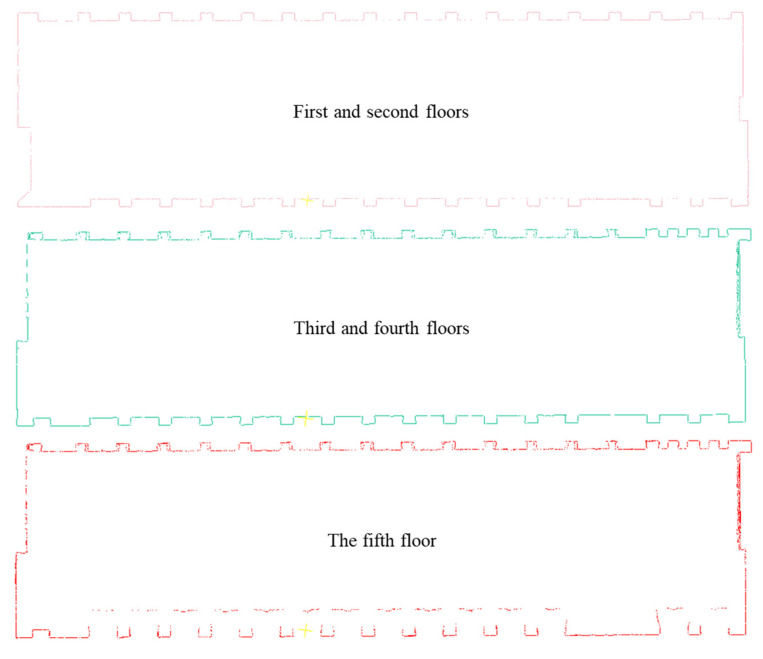
Results of the raw feature line extraction.

**Figure 13:**
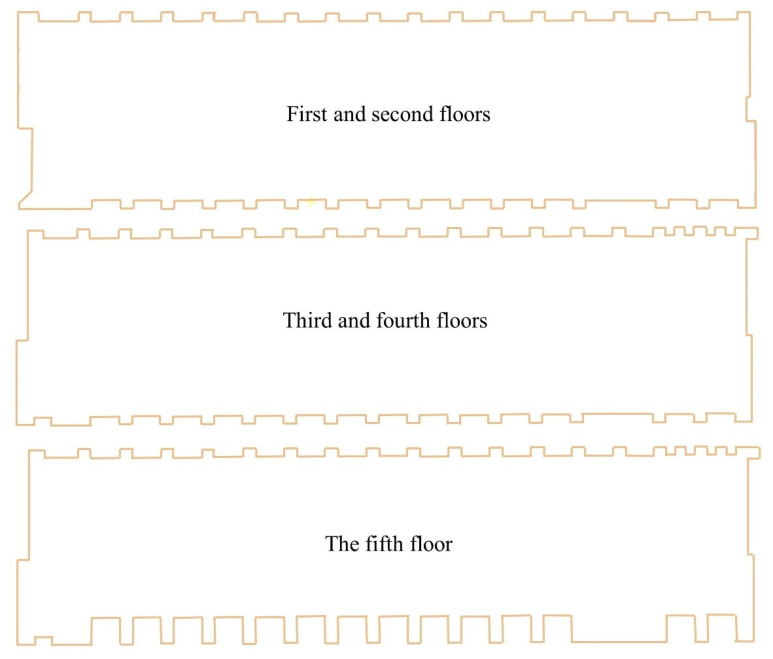
Results of topology reconstruction.

After obtaining the point cloud of the No. 36 building, the slicing method was employed to obtain point cloud slices for extracting wall feature lines. The methods proposed in Section 3.2.2 were used to extract wall feature lines from slice clouds at different elevations. The extraction and topology reconstruction results of the feature lines are shown in Figure 12 and Figure 13.

After organizing the extracted wall line features, an external program for automatically generating BIM models was developed based on the Revit API. The automated modeling program was written in C# within the Visual Studio 2022 environment. It transformed the point cloud of the No. 36 building into a parametric BIM model.

**Figure 14:**
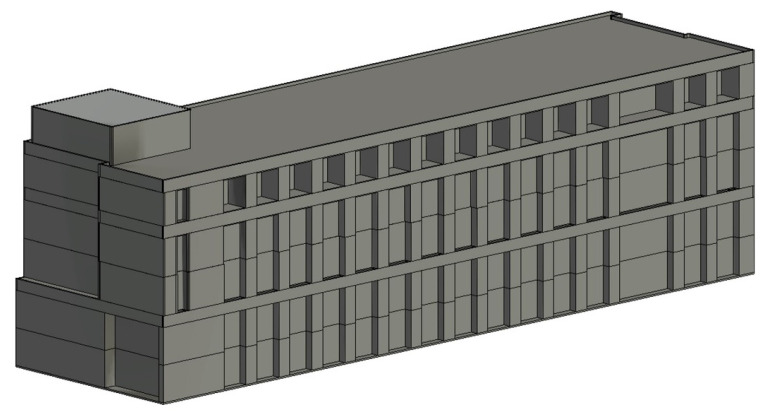
BIM object reconstruction result.

The selected wall type in our experiment is “conventional-250 mm”. By reading the exterior wall polylines, continuous wall solid objects are generated automatically using an external program. Since 3D reconstruction of the interior of the building was not conducted in this study, the reconstruction of the BIM model of the interior structure was not considered. Finally, the floor and ceiling are added to complete BIM object reconstruction. The results of the BIM model reconstruction are shown in Figure 14.

### 4.4. Defect Inspection and Visualization

**Figure 15:**
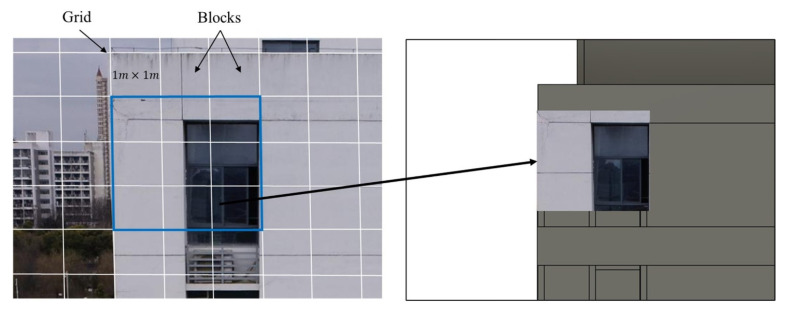
Cropping of drone image.

Crack semantic segmentation was taken as an example in the experiment to ensure transfer learning effectiveness using the pretrained K-Net semantic segmentation model. In addition to crack images selected from open-source datasets, additional building crack images were captured for training, inference validation, and model prediction. The image size of the open-source dataset is 224×224, and the additional captured images are 5472×3648. As Figure 15 shows, the additional captured images were also cropped to the grid, and those containing crack information were selected and added to the dataset.

The augmented dataset consisted of 400 crack images, encompassing cracks of various orientations, shapes, and distributions. All images were manually annotated using LabelMe to generate annotation files in mask format. Finally, the images and annotation files were divided into training, validation, and prediction sets at a ratio of 8:1:1.

Subsequently, the pretrained K-Net model was downloaded from the open-source library MMSegmentation [44] for semantic segmentation. The hardware configuration for the deep learning experiment is presented in Table 3. The training process was set to run for 20,000 iterations, with key metrics such as aAcc, mIoU, mAcc, mDice, mFscore, mPrecision, and mRecall calculated every 500 iterations to evaluate the performance of the model. Additionally, the model with the highest mIoU was selected and saved as the best model weight file every 2500 iterations. The transfer training process took about 6 h.

**Figure 16:**
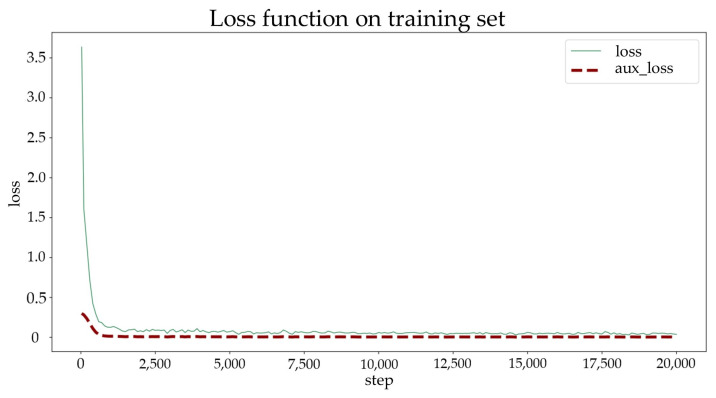
Loss function on the training set.

**Figure 17:**
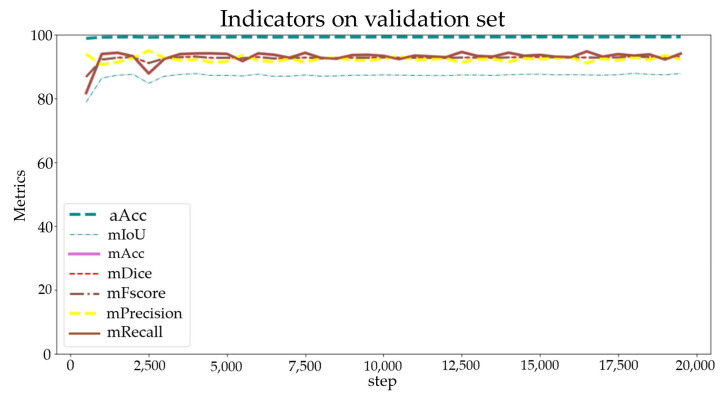
Indicators on the validation set.

**Figure 18:**
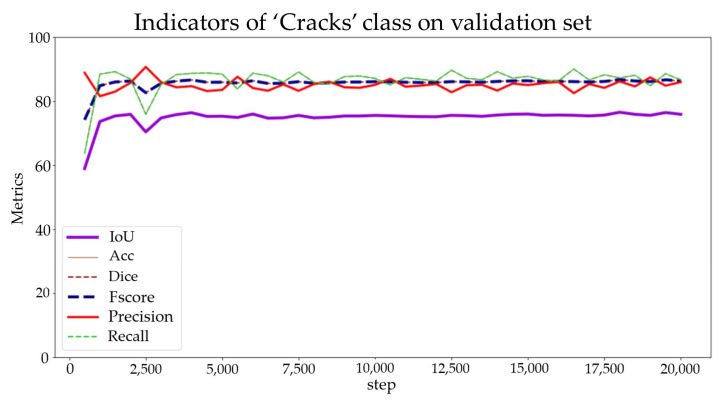
Indicators of the “crack” labels on the validation set.

The loss function and auxiliary loss function of the K-Net pretrained model on the training set are illustrated in Figure 16. Within the first 800 iterations, a rapid decline occurs in the loss function gradient. From the 800th to the 2500th iteration, the gradient descent gradually becomes smoother; it stabilizes after the 2500th iteration. The initial learning rate for the training process was set to $2\times 10^{-6}$ and gradually increased to $6\times 10^{-5}$ within 1000 iterations. Additionally, the average metric parameters during the validation process and the individual metric parameters for the “crack” label are presented in Figure 17 and Figure 18, respectively.

As Figure 16 shows, after approximately 3000 iterations, the IoU metric stabilizes at approximately 75, while the scores for the other metrics fluctuate at approximately 85. Throughout the learning process, there were no instances of overfitting or underfitting. Additionally, the optimal weight model was obtained at the 18,000th iteration. The trained model demonstrates satisfactory performance and can be effectively utilized for crack semantic segmentation tasks.

**Figure 19:**
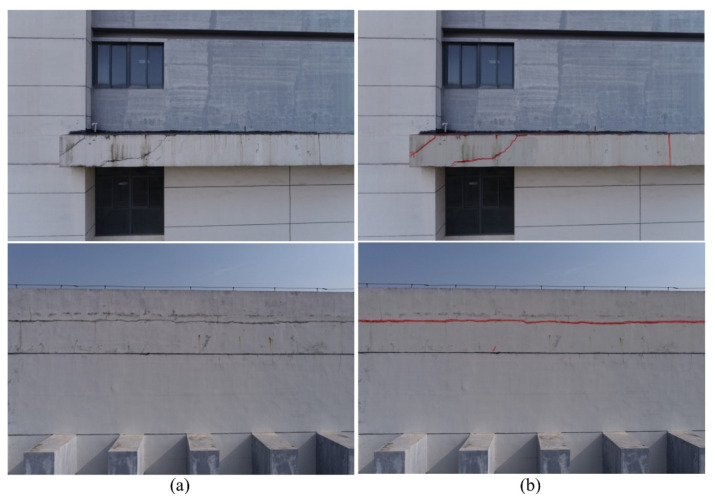
Semantic segmentation results. (**a**) Original images; (**b**) semantic segmentation overlay on original images.

Finally, inference prediction is conducted using the newly trained model to assess the semantic segmentation accuracy and generalization capability, resulting in a semantic segmentation map of cracks on building surfaces (Figure 19).

### 4.5. Result of Creating the SDE-BIM

**Figure 20:**
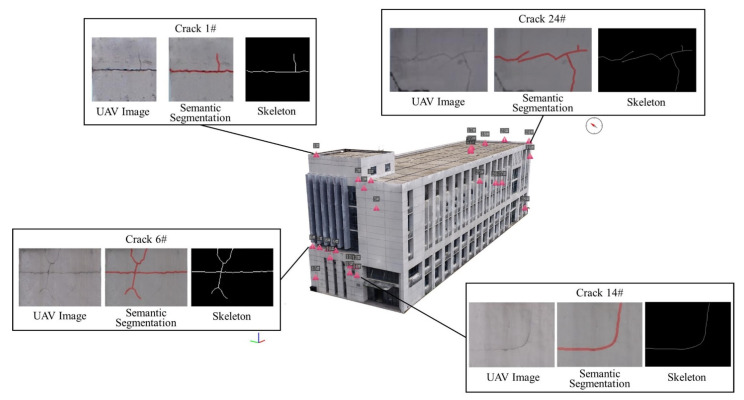
Result of creating the SDE-BIM.

The created surface defect-extended BIM is exhibited in Figure 20. Partial results of the calculated geometry feature parameters are subsequently calculated. The BIM model and defect inspection information is integrated into one model. The model can be dynamically updated according to the building defect inspection results obtained during different periods. Compared to static ordinary BIM models, the created model can store a large amount of building information and reflect the actual health status of the building, which is beneficial for building maintenance.

The length and width calculation results presented in Section 3.4 are all in pixels. If it is necessary to calculate the real-world length and width, the conversion should be based on the conversion relationship between the image, camera, and real-world coordinate systems. According to this rule, partial results of the length and width calculations are shown in Table 4 and Table 5. Partial results of the geometric moment calculations are listed in Table 6. More inspection results can be found in Figures S1 and S2 in the Supplementary Materials.

Finally, we made an approximate estimate of the time consumption for the facade inspection experiment. Taking the south facade as an example, the area of the south facade was $75\times 22\;m^{2}$, and 105 images were captured using ortho-to-facade photography. In the post-processing stage, our research focused on acquiring accurate defect coordinates on the BIM project coordinate system using a texture mapping-based method. It contains four steps, including image preprocessing, crack prediction, texture mapping, and geometric parameter calculating. Excluding the delay caused by manual interference, the estimation of time consumption for each stage of the defect inspection of the south facade is shown in Table 7.

Due to the multiple subprocesses and manual factors in the experiment, it is difficult to accurately calculate the time consumption. The time consumption is estimated approximately. In image preprocessing stage, nine blocks were generated for each UAV image according to the rules in Figure 15. The block images were not generated in real time. The image preprocessing mainly includes camera pose extraction and block image generation, which takes about 15 min. Then, we spent time performing crack detection. The crack detection time for each block during the model prediction process was approximately 0.5 s and the whole time was 14 min (regardless of the presence of cracks). The defect block images were mapped to the BIM coordinate system, and each defect block image took 1–2 s to complete the texture mapping. It took 42 min to map the entire south facade. The final step was geometric parameter calculating. The time spent calculating the length, width, and geometric moments of a crack was within 0.53 s on average and the total time cost was 2 min. The total time spent on the south facade inspection was 73 min.

## 5. Discussion

In the experiment, dense point clouds were reconstructed from UAV oblique images to generate a topological BIM model; defect information was detected using a K-Net neural network, and defect information modeling was successfully integrated into the BIM model. The proposed method has significant advantages in reconstructing buildings from point clouds with heavy occlusions and missing data. Our defect detection method has some basic demands for input data sources.

**Figure 21:**
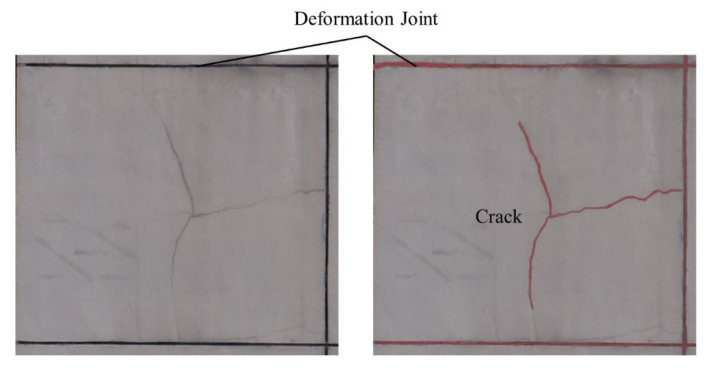
Deformation joints that are incorrectly detected as cracks.

When drones capture surface defects on buildings, they should be as close to the surface as possible to obtain sufficiently clear surface crack images. Additionally, an ortho-to-facade shooting method is adopted to ensure that the segmented images meet the requirements of neural network training and validation while accurately mapping to the grid on the surface of the BIM model. In the facade defect inspection stage, some inherent deformation joints on the walls can also be mistakenly identified as cracks because their geometric characteristics resemble defect cracks (as shown in Figure 21). Existing approaches still have difficulty distinguishing cracks from deformation joints. Additionally, several shadow regions on the images are likely to be identified as cracks. It is necessary to avoid the period of oblique sunlight when conducting aerial photography. For stains on the facades, a feasible method is to add a stain label to the dataset and participate in the training of the deep learning models.

Moreover, the inspection objects of this study were two-dimensional facade defects of buildings, which did not explore the true situation of the defects. These defects are three-dimensional, with cracks, patches, and peeling all having depth. The modeling of 3D defects still requires further research.

## 6. Conclusions

In this article, a framework for creating building surface defect information models based on drone images and deep learning is proposed. UAV images were acquired for BIM reconstruction and defect detection. A topologically consistent BIM model was reconstructed from dense SfM point clouds. A training dataset for defect detection was created from UAV images, and the trained model was applied to model the No. 36 teaching building at Nantong University. A texture mapping-based approach was employed to project defect geometry onto the surface of the BIM model and successfully generated an SDE-BIM. The proposed framework for creating building surface defect information models is applicable to most civil building and architectural inspection scenarios, offering a new solution for future large-scale building inspection tasks and architectural information storage.

However, the reconstruction of BIM objects for interior building scenes and multiple buildings is not considered in this work. In the future, we will combine UAV-based 3D reconstruction with TLS [45] to generate complete building point clouds and extract building structural parameters for BIM modeling. With the experience gained in this study, we will make further attempts to resolve the problem of constructing defect information models for building complexes. A real-time defect detection system will be further developed to improve the efficiency and reliability of the system.

## Figures and Tables

**Table 3:** Hardware configuration for deep learning.

Hardware/Environment	Types/Parameters
CPU	Intel Xeon E5-2680 v4 @ 2.40 GHz
RAM	32 G
GPU	NVIDIA RTX A4000 16 G
Framework	PyTorch 2.0.1
PythonVersion	3.10.12

**Table 4:** Partial results of the length calculation.

Crack ID	Absolute Length/mm	Actual Length/mm
1#	11,010	12,130
6#	1680	3670
14#	950	1240
24#	830	1930

**Table 5:** Partial results of the width calculation.

Crack ID	Mean Width/mm	Maximum Width/mm
1#	13	18
6#	12	16
14#	9	11
24#	10	12

**Table 6:** Partial results of the geometric moment calculation.

Crack ID	Zero-Order Moment	First-Order Moments	Second-Order Moments
1#	5384	$m_{10}=$ 10,778,595.0 $m_{01}=$ 9,939,390.0	$m_{20}=$ 178,700,504.7 $m_{02}=$ 11,734,371.5 $m_{11}=$ −13,448,553.8
6#	945	$m_{10}=$ 16,308,780.0 $m_{01}=$ 10,965,765.0	$m_{20}=$ 278,975,152.3 $m_{02}=$ 106,254,475.6 $m_{11}=$ −16,014,058.8
14#	432	$m_{10}=$ 16,601,010.0 $m_{01}=$ 14,334,315.0	$m_{20}=$ 291,822,112.2 $m_{02}=$ 152,243,606.0 $m_{11}=$ −151,858,244.0
24#	546	$m_{10}=$ 28,562,295.0 $m_{01}=$ 16,840,455.0	$m_{20}=$ 710,940,141.6 $m_{02}=$ 91,471,456.7 $m_{11}=$ 49,305,995.3

**Table 7:** The time consumption for each phase (south facade).

Process Phase	Time Consumption
Image preprocessing (min)	15
Crack prediction (min)	14
Defect block image texture mapping (min)	42
Geometric parameter calculating (min)	2
Total time (min)	73

## Data Availability

The raw data supporting the conclusions of this article will be made available by the authors on request.

## Supplementary Materials

The following supporting information can be downloaded at: https://www.mdpi.com/article/10.3390/s24134151/s1, Figure S1: Semantic segmentation and binarization results of cracks 1# to 5#; Figure S2: Extraction results of crack edges and skeletons of cracks 1# to 5#; Table S1: Facade crack information; Table S2: Calculation result of crack length; Table S3: Calculation result of crack width; Table S4: Calculation result of geometry moment.

## Appendix A

The proposed feature line extraction algorithm based on eigenvectors is as follows:

(1) Rasterizing building point cloud slices: According to the input sliced point cloud $P_{slice}$ and pixel size $S_{pixel}$, the coordinate range of the point cloud and the number of rows and columns of the occupied raster image s after projecting the slice are calculated first. The point cloud is traversed, the row and column numbers in the raster image are calculated, and 1 is added to the number of points for the pixels in the corresponding row and column to obtain the occupied raster image.

(2) Calculating the weighted centroid points: A KD-tree tree1 is created for the point cloud slice $P_{slice}=\left\{P_{1},P_{2},\cdots ,P_{i}\right\},\;P_{i}=\left(x_{i},\;y_{i},0\right)$ based on the input pixel size $S_{pixel}$ and noise level S. Each pixel in the raster image is upsampled, and each pixel is subdivided into 5×5 subpixels. Whether each subdivided pixel contains sample points is determined by searching for the number of neighbors around the center point of each subdivided pixel. If neighboring points exist within the same radius range as $S_{pixel}$, the subdivided pixel is added to the set $G_{gravity}$. Traverse $G_{gravity}$, set the search radius of tree1 to $S+S_{pixel}$, and calculate the weighted centroid points of the subdivided pixels with sample points using Formula (A1).

(A1) $$X_{i+1}=\frac{\sum_{j\in J}X_{i}W_{j}}{\sum_{j\in J}W_{j}},\;{where\;W}_{j}=\frac{1}{\left\|x_{i}-q_{i}\right\|}$$

(3) Iterative refinement: A new KD-tree tree2 is created for the set of weight central points $P_{weight}$, and an improved mean-shift algorithm [46,47] (Formula (A2)) is used for calculating the new positions of the central points.

(A2) $$x_{i}^{k+1}=\frac{\sum_{j\in J}P_{j}^{k}\alpha_{ij}^{k}}{\sum_{j\in J}\alpha_{ij}^{k}}+\lambda \frac{\sum_{i^{\prime }\in I\{i\}}(x_{i}^{k}-x_{i^{\prime }}^{k})\beta_{{ii}^{\prime }}^{k}}{\sum_{i^{\prime }\in I\{i\}}\beta_{{ii}^{\prime }}^{k}}$$

In Formula (A2), the first term is calculated by searching the neighborhood $N_{1}$ of the slice using tree1, and the second term is calculated by searching the neighborhood $N_{2}$ of the optimized weighted centroids set using tree2. The iteration parameter is an optional parameter with a default value of 3. The balancing parameter λ has a default value of 0.35.

(4) Feature calculation: The KD-tree tree2 is reconstructed for the optimized weighted central point set, the central points are rasterized, and each pixel is traversed and associated with the weighted central point. When multiple points fall into one pixel, one point is arbitrarily selected as the pixel’s associated central point. Then, the eigenvalues and eigenvectors of each pixel are calculated, and the eigenvector corresponding to the largest eigenvalue is recorded as the directional vector. The direction vector is subsequently converted to a quadrant angle. According to the eigenvalues, the curvature of each pixel is calculated and stored in quadrant angle raster images and curvature raster images.

(5) Iterative growth ensuring precision: First, pixels are sorted based on curvature. Pixels with smaller curvatures are more likely to be straight lines, so the pixel with the smallest curvature is selected as the seed point. Next, a 5×5 sliding window around the seed point is searched to determine whether the direction vector values of the neighboring points are the same as those of the current point. Iterative growth is conducted by evaluating the similarity of all neighboring pixels Q to each seed pixel in the seed unit set. Then, the algorithm iterates through all neighboring pixels. If the direction angle $\theta_{j}$ between the current pixel and its neighbor $q_{i}$ is less than the threshold, namely, $\theta_{j}<\Delta \theta$, all points in the pixel unit are added to the set Ψ, and the angle of the line support region is updated. The pixel is then regarded as a new growth center, and the similarity of its neighboring pixels is evaluated. Once a pixel is added to a region, it is labeled and will not be visited again. The above process is repeated until all neighboring pixels have been traversed. For set Ψ, the bounding box is computed; the line support region (a group of pixels) must be associated with a line segment (essentially a rectangular area). The line segment is determined by its endpoints and width, or by its center, angle, length, and width. The parameters of the feature lines are extracted according to the rectangular region, thus defining a feature line segment $L_{i}$. Finally, the above steps are repeated. New seed points are selected for iteration until all seed points are traversed, and the final set of feature lines $L=\left\{L_{1},L_{2},\cdots ,L_{n}\right\}$ is obtained.
