# Supplementary material for: Surface Defect-Extended BIM Generation Leveraging UAV Images and Deep Learning
Source: Sensors (Basel). 2024 Jun 26;24(13):4151. doi: 10.3390/s24134151 (PMC11243814; doi:10.3390/s24134151)
Supplement: Supplementary file 1 [file sensors-24-04151-s001.zip › sensors-3035617-supplementary.pdf]

Generate Surface Defect Extended BIM Leveraging UAV Images and Deep Learning Supplementary Material

Lei Yang [1, 2], Keju Liu [3], Ruisi Ou [3], Peng Qian [2, 4, *], Yunjie Wu [2], Zhuang Tian [2], Changping Zhu [4], Sining Feng [4], and Fan Yang [1, 3, 4, *]

The document provides additional experimental results, algorithmic details, which were not included in the main paper due to the space limitation.

In Section 4.5, before calculating the geometric parameters of cracks, some image processing works are required. In this section, taking crack 1# to 5 # as examples, the semantic segmentation and binarization results are shown in Figure 1. In Figure 2, the extracted crack edges and skeletons are presented.

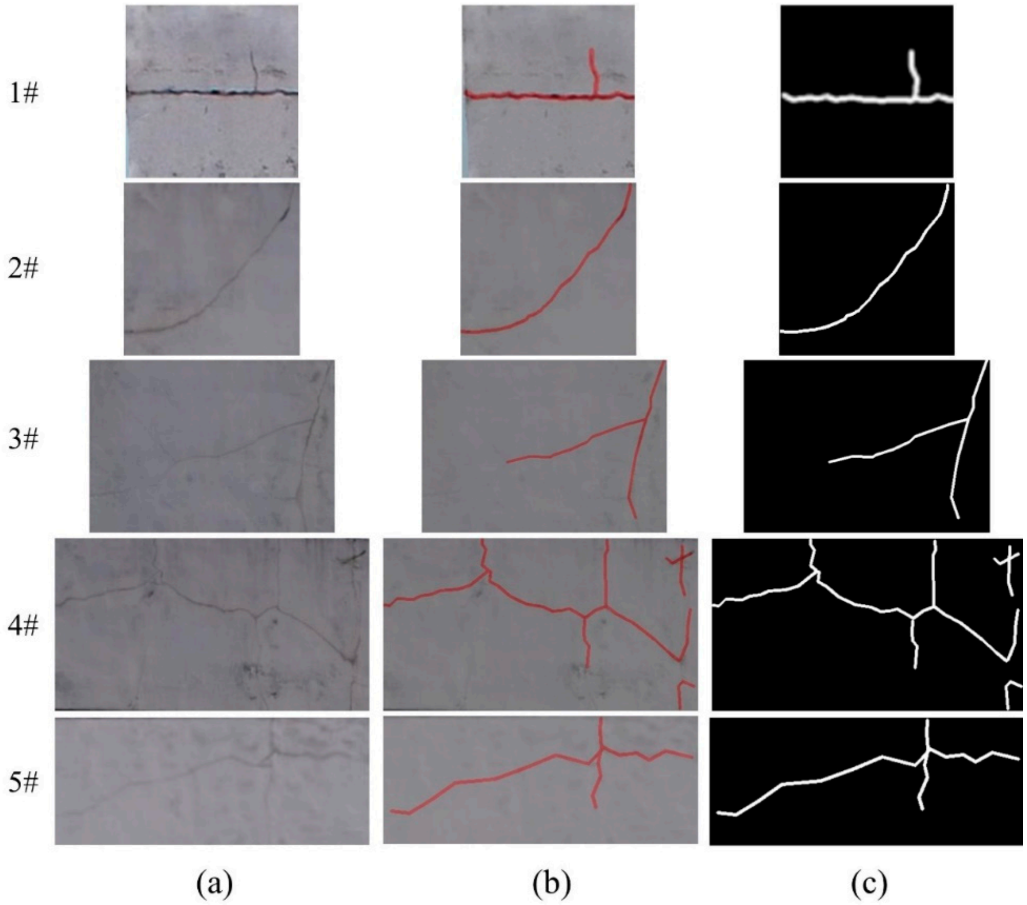

Figure S1. Semantic segmentation and binarization results of cracks 1# to 5#. (a) raw images; (b) semantic segmentation results; (c) binary images.

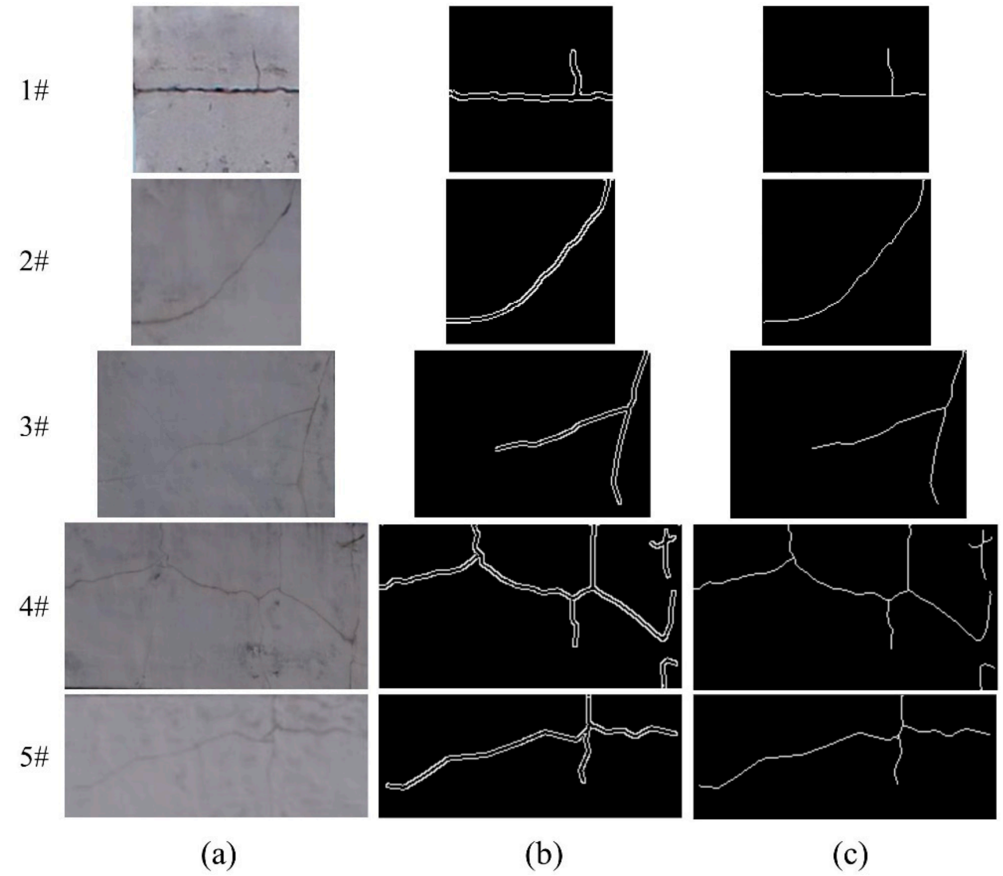

Figure S2. Extraction results of crack edges and skeletons of cracks 1# to 5#. (a) raw images; (b) crack edges; (c) crack skeletons.

Then, according to the geometric parameter calculation method introduced in Section 3.4, the length, width and geometric moment of all detected facade cracks were calculated. The basic information of cracks is shown in Table 1, and all calculation results are listed in Tables 2, 3, and 4.

(1) Facade crack information. (Crack 1# to 28#)

Table S1. Facade crack information.

Number	Geometry feature	Position
1#	Horizontal crack	Equipment room on roof
2#	Oblique crack	West facade on 5th floor
3#	Branching crack	West facade on 5th floor
4#	Branching crack	West facade on 5th floor
5#	Cross crack	West facade on 5th floor
6#	Branching crack	West facade on 2nd floor
7#	Cross crack	West facade on 2nd floor
8#	Cross crack	West facade on 2nd floor
9#	Oblique crack	West facade on 2nd floor
10#	Reticular crack	West facade on 2nd floor
11#	Oblique crack	West facade on 2nd floor

12#	Oblique crack	West facade on 2nd floor
13#	Branching crack	West facade on 1st floor
14#	Oblique crack	West facade on 1st floor
15#	Branching crack	West facade on 1st floor
16#	Branching crack	South facade on 4th floor
17#	Branching crack	East facade on 5th floor
18#	Horizontal crack	East facade on 5th floor
19#	Oblique crack	East facade on 5th floor
20#	Horizontal crack	East facade on 5th floor
21#	Horizontal crack	East facade on 5th floor
22#	Horizontal crack	East facade on 5th floor
23#	Reticular crack	East facade on 5th floor
24#	Branching crack	East facade on 5th floor
25#	Vertical crack	East facade on 2nd floor
26#	Oblique crack	East facade on 2nd floor
27#	Oblique crack	East facade on 2nd floor
28#	Branching crack	East facade on 1st floor

(2) Calculation result of crack length.

Table S2. Calculation result of crack length.

Number	Absolute length/mm	Real length/mm
1#	11010	12130
2#	1330	1420
3#	Main branch: 1460	2620
4#	Main branch: 2350	5820
5#	Horizontal: 2580; Vertical: 790	3530
6#	Main branch:1680	3670
7#	Horizontal: 2010; Vertical:1440	3540
8#	Horizontal: 1970; Vertical:1420	3620
9#	1080	1410
10#	/	2780
11#	870	1010
12#	860	1120
13#	Main branch:1170	2790
14#	950	1240
15#	Main branch:1010	1850
16#	Main branch:870	3640
17#	Main branch:1210	3270
18#	7480	7680

19#	1060	1010
20#	870	910
21#	740	760
22#	840	860
23#	/	5150
24#	Main branch:830	1930
25#	920	920
26#	2330	2770
27#	900	1030
28#	/	4970

(3) Calculation result of crack width.

Table S3. Calculation result of crack width.

Number	Mean width/mm	Maximum width/mm
1#	13	18
2#	8	15
3#	8	10
4#	9	12
5#	8	10
6#	12	16
7#	9	11
8#	9	12
9#	7	11
10#	8	10
11#	12	15
12#	11	13
13#	8	9
14#	9	11
15#	8	12
16#	7	9
17#	15	20
18#	9	16
19#	9	12
20#	8	10
21#	9	13
22#	8	9
23#	10	14
24#	10	12
25#	13	15

26#	10	20
27#	11	18
28#	9	13

(4) Calculation result of geometry moment.

Table S4. Calculation result of geometry moment.

Number	Zero-order moment	First-order moments	Second-order moments
1#	5384	m_{10} =10778595.0<br>m_{01} =9939390.0	m_{20} =178700504.7<br>m_{02} =11734371.5<br>m_{11} =-13448553.8
2#	489	m_{10} =8205900.0<br>m_{01} =6281670.0	m_{20} =155529582.3<br>m_{02} =203191335.0<br>m_{11} =-91206265.6
3#	429	m_{10} =14610225.0<br>m_{01} =6142950.0	m_{20} =101712860.5<br>m_{02} =66070970.8<br>m_{11} =-32720226.9
4#	1248	m_{10} =42459540.0<br>m_{01} =12509535.0	m_{20} =1309954532.3<br>m_{02} =278362628.4<br>m_{11} =217891044.6
5#	725	m_{10} =21898380.0<br>m_{01} =7052790.0	m_{20} =591752340.2<br>m_{02} =41894947.5<br>m_{11} =-117418463.5
6#	945	m_{10} =16308780.0<br>m_{01} =10965765.0	m_{20} =278975152.3<br>m_{02} =106254475.6<br>m_{11} =-16014058.8
7#	787	m_{10} =13476750.0<br>m_{01} =9503340.0	m_{20} =348858461.1<br>m_{02} =112251023.3<br>m_{11} =-20410428.4
8#	713	m_{10} =13595580.0<br>m_{01} =8677140.0	m_{20} =219119343.4<br>m_{02} =85749390.8<br>m_{11} =-12749415.6
9#	421	m_{10} =17967810.0<br>m_{01} =12311655.0	m_{20} =359406681.5<br>m_{02} =94660379.5<br>m_{11} =-129695379.8
10#	1285	m_{10} =30625755.0<br>m_{01} =17546295.0	m_{20} =349813989.7<br>m_{02} =217618608.2<br>m_{11} =-30844168.8
11#	314	m_{10} =8077380.0<br>m_{01} =8469825.0	m_{20} =107856457.7<br>m_{02} =90317835.9<br>m_{11} =85914681.3
12#	497	m_{10} =13712880.0<br>m_{01} =12356535.0	m_{20} =449144234.6<br>m_{02} =71685249.3

			m_{11} =147751602.8
13#	699	m_{10} =32214405.0<br>m_{01} =19796670.0	m_{20} =390394403.5<br>m_{02} =273800135.1<br>m_{11} =41834602.4
14#	432	m_{10} =16601010.0<br>m_{01} =14334315.0	m_{20} =291822112.2<br>m_{02} =152243606.0<br>m_{11} =-151858244.0
15#	559	m_{10} =10168380.0<br>m_{01} =8252565.0	m_{20} =109244153.1<br>m_{02} =83009837.1<br>m_{11} =-9330517.1
16#	828	m_{10} =21064020.0<br>m_{01} =13628985.0	m_{20} =325825639.0<br>m_{02} =107675527.3<br>m_{11} =47834864.9
17#	1470	m_{10} =67125435.0<br>m_{01} =66578970.0	m_{20} =309602464.0<br>m_{02} =55856195.0<br>m_{11} =79338956.8
18#	1941	m_{10} =335775585.0<br>m_{01} =27156735.0	m_{20} =387245546.8<br>m_{02} =199750005.1<br>m_{11} =-149101149.9
19#	592	m_{10} =35262930.0<br>m_{01} =16109115.0	m_{20} =125192958.0<br>m_{02} =57413270.3<br>m_{11} =-231485918.4
20#	638	m_{10} =32685645.0<br>m_{01} =12292785.0	m_{20} =105913470.8<br>m_{02} =9315325.2<br>m_{11} =-71472570.6
21#	509	m_{10} =47235435.0<br>m_{01} =13832985.0	m_{20} =1391694448.3<br>m_{02} =3576628.5<br>m_{11} =-21881087.2
22#	455	m_{10} =18060630.0<br>m_{01} =9757830.0	m_{20} =491386179.9<br>m_{02} =9451936.2<br>m_{11} =-64513448.7
23#	1323	m_{10} =51394740.0<br>m_{01} =61127835.0	m_{20} =1015401992.9<br>m_{02} =1196818117.0<br>m_{11} =145060888.7
24#	546	m_{10} =28562295.0<br>m_{01} =16840455.0	m_{20} =710940141.6<br>m_{02} =91471456.7<br>m_{11} =49305995.3
25#	570	m_{10} =19988175.0<br>m_{01} =27416580.0	m_{20} =172732089.4<br>m_{02} =824505834.2<br>m_{11} =1061153.7
26#	967	m_{10} =75677370.0<br>m_{01} =33367515.0	m_{20} =4251790142.5<br>m_{02} =992927274.6<br>m_{11} =-918381702.0
27#	316	m_{10} =8128125.0	m_{20} =199128889.7

		m_{01} =8852070.0	m_{02} =125061402.2<br>m_{11} =-151337052.1
28#	1254	m_{10} =81982500.0<br>m_{01} =44352150.0	m_{20} =2106673442.7<br>m_{02} =1398817105.9<br>m_{11} =141711110.1
